# Thrombin Induces COX-2 and PGE_2_ Expression via PAR1/PKCalpha/MAPK-Dependent NF-kappaB Activation in Human Tracheal Smooth Muscle Cells

**DOI:** 10.1155/2022/4600029

**Published:** 2022-04-19

**Authors:** Chien-Chung Yang, Li-Der Hsiao, Ya-Fang Shih, Chih-Kai Hsu, Chia-Yu Hu, Chuen-Mao Yang

**Affiliations:** ^1^Department of Traditional Chinese Medicine, Chang Gung Memorial Hospital at Tao-Yuan, Kwei-San, Tao-Yuan 33302, Taiwan; ^2^School of Traditional Chinese Medicine, College of Medicine, Chang Gung University, Kwei-San, Tao-Yuan 33302, Taiwan; ^3^Department of Pharmacology, College of Medicine, China Medical University, Taichung 40402, Taiwan; ^4^Ph.D. Program for Biotech Pharmaceutical Industry, China Medical University, Taichung 40402, Taiwan; ^5^Department of Post-Baccalaureate Veterinary Medicine, College of Medical and Health Science, Asia University, Wufeng, Taichung 41354, Taiwan

## Abstract

The inflammation of the airway and lung could be triggered by upregulation cyclooxygenase (COX)-2 and prostaglandin E_2_ (PGE_2_) induced by various proinflammatory factors. COX-2 induction by thrombin has been shown to play a vital role in various inflammatory diseases. However, in human tracheal smooth muscle cells (HTSMCs), how thrombin enhanced the levels of COX-2/PGE_2_ is not completely characterized. Thus, in this study, the levels of COX-2 expression and PGE_2_ synthesis induced by thrombin were determined by Western blot, promoter-reporter assay, real-time PCR, and ELISA kit. The various signaling components involved in the thrombin-mediated responses were differentiated by transfection with siRNAs and selective pharmacological inhibitors. The role of NF-*κ*B was assessed by a chromatin immunoprecipitation (ChIP) assay, immunofluorescent staining, as well as Western blot. Our results verified that thrombin markedly triggered PGE_2_ secretion via COX-2 upregulation which were diminished by the inhibitor of thrombin (PPACK), PAR1 (SCH79797), G_i/o_ protein (GPA2), G_q_ protein (GPA2A), PKC*α* (Gö6976), p38 MAPK (SB202190), JNK1/2 (SP600125), MEK1/2 (U0126), or NF-*κ*B (helenalin) and transfection with siRNA of PAR1, G_q_*α*, G_i_*α*, PKC*α*, JNK2, p38, p42, or p65. Moreover, thrombin induced PAR1-dependent PKC*α* phosphorylation in HTSMCs. We also observed that thrombin induced p38 MAPK, JNK1/2, and p42/p44 MAPK activation through a PAR1/PKC*α* pathway. Thrombin promoted phosphorylation of NF-*κ*B p65, leading to nuclear translocation and binding to the COX-2 promoter element to enhance promoter activity, which was reduced by Gö6976, SP600125, SB202190, or U0126. These findings supported that COX-2/PGE_2_ expression triggered by thrombin was engaged in PAR1/G_q_ or G_i/o_/PKC*α*/MAPK-dependent NF-*κ*B activation in HTSMCs.

## 1. Introduction

Tracheal smooth muscle regulates differences in ventilation as an end-response effector by contraction responding to proinflammatory mediators, exogenous substances released, and various neurotransmitters under pathological or homeostatic conditions [1]. In the airway of asthmatics, oversecretion of metabolites of lysophosphatidic acid and arachidonic acid (AA) have been discovered [2]. AA is further metabolized by cyclooxygenases (COX), such as the inducible COX-2 and the constitutive COX-1, to prostaglandins (PGs) [3]. Under basal conditions, COX-2 is extremely restricted, but it, as a mediator of inflammatory responses, is rapidly induced by various stimuli [3, 4]. Furthermore, an increase in COX-2 activity could enhance the synthesis of PGE_2_ in different kinds of cells [3, 5]. These pieces of research reveal that COX-2/PGE_2_ is essential for airway inflammation.

Thrombin is mainly responsible for coagulation in most broad studies. Thrombin is recognized to maintain homeostasis and modulate the coagulation cascade. In addition, it possesses pleiotropic effects which have been displayed only in *in vitro* studies: it displays a mitogenic activity on endothelial cells and smooth muscle cells and plays a role in inflammation [6]. The hormone-like actions on cells by thrombin are mediated through one kind of protease-activated receptors (PARs) coupled to various G-proteins [4]. There are four family members recovered, PAR-1 to -4. Thrombin has been shown to regulate respiratory diseases [7–9]. In addition, several pieces of research have also presented that COX-2 can be produced by thrombin in human lung fibroblasts [10] or primary human atrial fibroblasts [11]. However, in human tracheal smooth muscle cells (HTSMCs), the detailed mechanisms of thrombin-mediated expression of COX-2 are not entirely recognized. Therefore, clarifying the mechanisms of how COX-2 is produced by thrombin may be helpful to approach the therapeutics of lung diseases.

In chronic airway diseases like COPD and asthma, one of the important signaling intermediates is PKC. PKC*α* has been linked to mucous production, spasm of the bronchus, and airway inflammation [12]. MAPKs also are essential signaling components triggered by growth factors, mechanical and chemical stressors, neurotransmitters, as well as cytokines [13]. In the airways, several signals lead to both acute and chronic responses which modulate contractile reaction and structure remodeling, respectively [14]. Thrombin has also been shown to activate JNK1/2 [15], p42/p44 MAPK [10, 16], and p38 MAPK [17] in various types of cells. However, whether these signalings could be activated by thrombin and participated in the expression of COX-2 leading to PGE_2_ release on HTSMCs remains unidentified. The COX-2 promoter possesses a variety of transcription factor binding elements, including NF-*κ*B and AP-1 [4]. NF-*κ*B displays as a master regulator in the phase of evolution and the resolution in inflammation [18, 19]; thus, it is recognized as a genius regulator of inflammatory responses. NF-*κ*B activation can be enhanced through a variety of signaling pathways, such as PKC*α* [5] or MAPKs [20]. Thus, in this study, we also challenged HTSMCs with thrombin to analyze the function of NF-*κ*B on the expression of COX-2.

The research was performed to study the impacts of thrombin on the expression of COX-2 leading to PGE_2_ release in HTSMCs. These data suggest that the upregulation of COX-2 expression associated with PGE_2_ release in thrombin-stimulated HTSMCs is mediated through PAR1/G_i/o_ or G_q_/PKC*α*/MAPK-dependent NF-*κ*B activation pathway.

## 2. Materials and Methods

### 2.1. Materials

Anti-phospho-JNK1/2, anti-phospho-p38 MAPK, anti-phospho-p42/p44 MAPK, and anti-phospho-p65 antibodies were ordered from Cell Signaling (Danvers, MA). Anti-lamin A, anti-COX-2, anti-PKC*α*, anti-GAPDH, anti-Gs*α*, anti-Gi*α*, anti-*β*-actin, anti-p42, anti-p38, anti-JNK2, and anti-p65 antibodies were ordered from Santa Cruz (Santa Cruz, CA). Tanshinone IIA, SP600125, GP antagonist-2A (GPA2A), PPACK, SCH79797, NS-398, GP antagonist-2 (GPA2), Gö6976, SB202190, U0126, helenalin, and celecoxib were purchased from Biomol (Plymouth Meeting, PA). SDS-PAGE supplies were bought from MDBio Inc. (Taipei, Taiwan). Other chemicals, enzymes, and thrombin (BRENDA: EC3.4.21.5, from bovine plasma, Catalog Number T4648) were obtained from Sigma-Aldrich (St. Louis, MO).

### 2.2. Cell Culture

HTSMCs purchased from ScienCell Research Laboratories are isolated from human trachea (San Diego, CA) and grown, as previously described [3]. The protocol for using the cell lines complied with the approval of Chang Gung University Institutional Animal Care and Use Committee (Approval Document No. CGU 16-046). Cells from passages 4 to 8 were used for experiments performed in this study. An XTT assay kit was used to determine the cytotoxic effects of DMSO and the concentrations of the inhibitors used in this study on these cells.

### 2.3. Western Blot Analysis

HTSMCs cultured onto 6-well plates were shifted to serum-free DMEM/F-12 medium overnight and then stimulated with thrombin for the indicated time points at 37°C. The whole-cell lysates were prepared by centrifuging at 45000 × *g* at 4°C for 10 min, as previously described with some modifications [3]. The denatured samples were separated by SDS-PAGE (10% running gel). After electrophoresis, the proteins were transferred to nitrocellulose membranes. To determine the levels of protein expression, respective primary antibodies were diluted with TTBS buffer in 1 : 1000, were added to membranes and incubated at 4°C for 24 h, and then, followed by a horseradish peroxidase-conjugated secondary antibody (1 : 1000) for 1 h. ECL reagents were adopted to examine the immunoreactive bands. The images of immunoblots were detected using a UVP BioSpectrum 500 imaging system (Upland, CA). The densities of immunoblots were scanned and analyzed by UN-SCAN-IT gel software (Orem, UT).

### 2.4. Measurement of Luciferase Promoter Activity of COX-2

A region spanning -459 to +9 bp of human COX-2 promoter was cloned into a pGL3-basic vector to construct the COX-2-luc plasmid. The cell lysates of HTSMCs treated with thrombin were prepared and determined the levels of promoter activity using a luciferase assay system (Promega, Madison, WI), as previously described with some modifications [3]. *β*-Gal activity was used as standardization for determining the firefly luciferase activities. The COX-2-luc plasmid was mutated by a mismatched mutation primer. A mt-NF-*κ*B primer, 5′-GGTAGGCTTACTGGGCCCCCAC-3′, was used to generate NF-*κ*B mt-luci which was applied to investigate the role of NF-*κ*B in COX-2 expression.

### 2.5. Total RNA Extraction and Real-Time PCR Analysis

HTSMCs were growth-arrested by shifting to serum-free DMEM/F-12 medium and incubated with thrombin for the indicated time points. RNA was harvested using TRIzol. The levels of COX-2 gene expression were measured using the TaqMan (GeneDireX®, San Diego, CA) gene expression assay system as previously described with some modifications [3]. All experiments were operated using the probe and primer mixes for *COX-2* and endogenous *GAPDH* control genes determined by a 7500 real-time PCR System (Applied Biosystems, Foster City, CA). The model of 2^(Cttestgene-CtGAPDH)^ (Ct = threshold cycle) was used to calculate the relative amount of the target gene. For amplification reaction, the following primers were employed:

COX-2:

Forward primer: 5′-CAAACTGAAATTTGACCCAGAACTAC-3′;

Reverse primer: 5′-ACTGTTGATAGTTGTATTTCTGGTCATGA-3′;

Probe: 5′-AACACCCTCTATCACTGGCATCCCCTTC-3′.

GAPDH:

Forward primer: 5′-GCCAGCCGAGCCACAT-3′;

Reverse primer: 5′-CTTTACCAGAGTTAAAAGCAGCCC-3′;

Probe: 5′-CCAAATCCGTTGACTCCGACCTTCA-3′.

### 2.6. Measurement of PGE_2_ Generation

While reaching confluence, HTSMCs cultured onto 6-well culture plates were challenged with thrombin at 37°C for the indicated time points. The levels of PGE_2_ released from HTSMCs were analyzed using a PGE_2_ ELISA kit (Cayman).

### 2.7. RT-PCR

RNA was extracted from HTSMCs using TRIzol. The expression of various target components was determined by RT-PCR, as previously described with some modifications [3]. cDNAs encoding PAR1, PAR2, PAR3, PAR4, and *β*-actin were amplified from 3 to 5 *μ*l of the cDNA mixed with reaction mixture using specific gene primers. The following primers were applied for the amplification:

*β*-Actin:

5′-GAACCCTAAGGCCAACCGTG-3′ (sense)

5′-TGGCATAGAGGTCTTTACGG-3′ (antisense)

PAR1:

5′-CAGTTTGGTCTGAATTGTGTCG-3′ (sense)

5′-TGCACGAGCTTATGCTGCTGAC-3′ (antisense)

PAR2:

5′-TGGATGAGTTTTCTGCATCTGTCC-3′ (sense)

5′-CGTGATGTTCAGGGCAGGAATG-3′ (antisense)

PAR3:

5′-TCCCCTTTTCTGCCTTGGAAG-3′ (sense)

5′-AAACTGTTGCCCACACCAGTCCAC-3′ (antisense)

PAR4:

5′-AACCTCTATGGTGCCTACGTGC-3′ (sense)

5′-CCAAGCCCAGCTAATTTTTG-3′ (antisense)

### 2.8. Transient Transfection with siRNAs

Human siRNAs of PAR1 (SASI_Hs01_00240436), G_q_ (SASI_Hs01_00148142), G_i_ (SASI_Hs01_00187237), PKC*α* (SASI_Hs01_00018815), p38 (SASI_Hs01_00027001), JNK2 (SASI_Hs01_00143828), p42 (SASI_Hs01_00124656), p65 (SASI_Hs01_00171090), and scramble were purchased from Sigma (St. Louis, MO). According to the instructions of the manufacturer, a Lipofectamine™ RNAiMAX reagent with 100 nM siRNAs was transiently transfected into the cells, as previously described with some modifications [5]. In brief, siRNA (100 nM) was formulated with Lipofectamine 2000 transfection reagent. The transfection mixture was diluted into 900 *μ*l of DMEM/F12 medium and directly added to the cells cultured onto 6-well plates. The cells were washed with PBS, replaced with serum-free DMEM/F-12 medium for 24 h, and then, treated with thrombin.

### 2.9. Chromatin Immunoprecipitation Assay

To detect the binding of transcription factor NF-*κ*B with human COX-2 promoter, chromatin immunoprecipitation (ChIP) analysis was conducted, as previously described [5]. Soluble chromatin was immunoprecipitated using anti-p65 antibody or normal goat immunoglobulin G (IgG). The immunoprecipitates were washed, eluted, and then, heated overnight at 65°C to recover DNA. Purified DNA fragments were analyzed on 2% agarose in 1x TAE gel containing ethidium bromide.

### 2.10. Isolation of Subcellular Fractions

“Cells were harvested, sonicated with a sonicator (Misonix Inc., Farmingdale, NY) for 5 s at output 1.5, and centrifuged for 15 min at 8000 rpm at 4°C. The pellet was collected as the nuclear fraction. The supernatant was centrifuged for 60 min at 14000 rpm at 4°C to yield the supernatant (cytosolic fraction) and the pellet (membrane fraction), as previously described with some modifications [5].”

### 2.11. Statistical Analysis of Data

The data were statistically analyzed using a GraphPad Prism Program 6.0 software (GraphPad, San Diego, CA), as previously described [5]. “We used one-way ANOVA followed by Dunnett's post hoc test when comparing more than two groups of data or nonparametric Kruskal–Wallis test, followed by Dunn's post hoc test, post hoc tests were run only if there was no significance in the homogeneity of variance and *F* achieved *P* < 0.05. *P* values of 0.05 were considered to be statistically significant. All the data were expressed as the mean ± SEM, at least three individual experiments (*n* = number of independent cell culture preparations).”

## 3. Results

### 3.1. Thrombin Induces COX-2 Expression and PGE_2_ Release via PAR1

**Figure 1:**
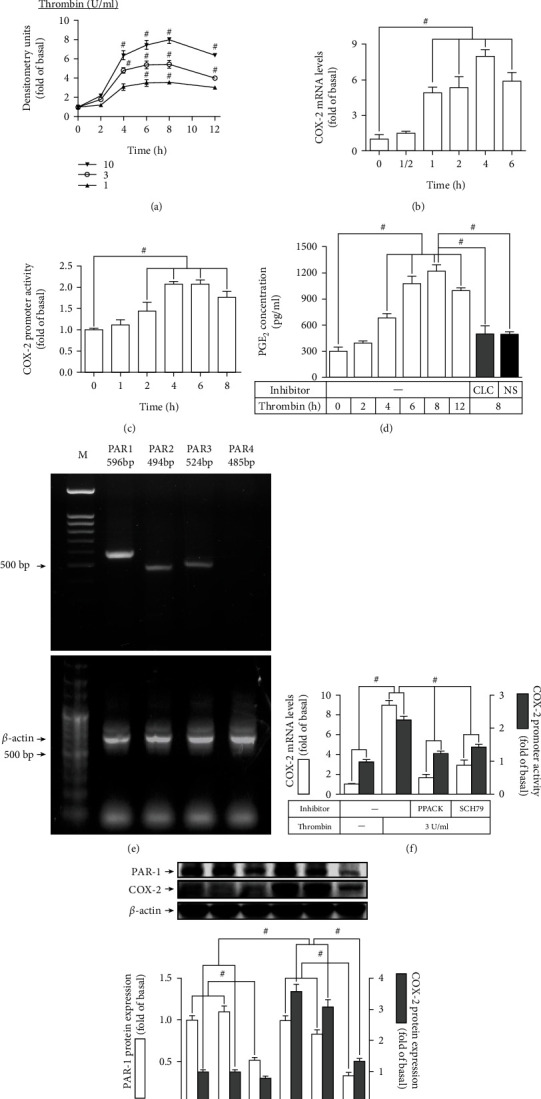
Thrombin induces COX-2 expression via PAR1. (a) Cells were treated with thrombin (1, 3, or 10 U/ml) for the indicated time intervals. The protein expression of COX-2 was determined by Western blot. (b, c) Cells were treated with 3 U/ml thrombin for the indicated time intervals. The COX-2 (b) mRNA levels and (c) promoter activity were determined by real-time PCR and promoter assay, respectively. (d) Cells were treated with 3 U/ml thrombin for the indicated time intervals or pretreated with 10 *μ*M CLC or 10 *μ*M NS-398 for 1 h and then treated with thrombin for 8 h. The PGE_2_ generation was measured. (e) The levels of *PAR1*, *PAR2*, *PAR3*, *PAR4*, and *β-actin* (as an internal control) mRNA on HTSMCs were determined by RT-PCR. (f) Cells were pretreated with PPACK (3 *μ*M) or SCH79797 (10 *μ*M) for 1 h and then incubated with thrombin for 4 h. The mRNA levels and promoter activity of COX-2 were determined. (g) Cells were transfected with either scrambled or PAR1 siRNA and then incubated with thrombin for 6 h. The protein levels of PAR1 and COX-2 were determined by Western blot. Data are expressed as mean ± S.E.M. of three independent experiments. ^#^*P* < 0.05, as compared with the control or pretreatment with inhibitor indicated in the figure.

First, in HTSMCs, we examined the levels of COX-2 produced by thrombin.  Figure 1(a) showed that thrombin upregulated protein levels of COX-2 which were dependent on its concentrations and incubation time. Besides, the amount of *COX-2* mRNA expression was time-dependently enhanced by thrombin (3 U/ml) for the time intervals as indicated. Data in Figure 1(b) demonstrated that thrombin time-dependently increased the expression of *COX-2* mRNA reaching a maximal level at 4 h during the period of incubation in HTSMCs. Furthermore, 3 U/ml thrombin time-dependently enhanced promoter activity of COX-2 with the highest activity at 4 h (Figure 1(c)). PGE_2_ which is converted from AA by COX-2 could be applied as an indicator of the activity of COX-2. In addition, in this study, our findings showed that thrombin time-dependently produced PGE_2_ secretion reaching a maximal amount within 8 h, which was diminished by COX-2 inhibitors (NS-398 and celecoxib) (Figure 1(d)). Thrombin can activate PARs leading to the changes in cellular functions on various types of cells. Thrombin can directly trigger PAR1, PAR3, and PAR4, but not PAR2 which was activated by trypsin [21]. Actually, our findings showed that PAR1-3 were expressed on HTSMCs (Figure 1(e)). Thus, we hypothesized that thrombin could mediate through either PAR-1 or -3 to enhance COX-2 upregulation accompanied with PGE_2_ release in HTSMCs. Here, we found that pretreatment with PPACK (an irreversible thrombin inhibitor) or SCH79797 (a selective PAR1 antagonist) significantly reduced thrombin-induced promoter activity and mRNA levels of COX-2 (Figure 1(f)). We additionally applied PAR1 siRNA to verify the function of PAR1 in the levels of COX-2 protein stimulated by thrombin. Both PAR1 protein level and the expression of COX-2 stimulated by thrombin were reduced in HTSMCs transfected with PAR1 siRNA (Figure 1(g)). Altogether, these discoveries in HTSMCs suggested that PAR1 participates in the thrombin-provoked COX-2/PGE_2_ upregulation.

### 3.2. Thrombin Enhances Expression of COX-2 via G_q_ and G_i/o_ Protein-Coupled Receptors

**Figure 2:**
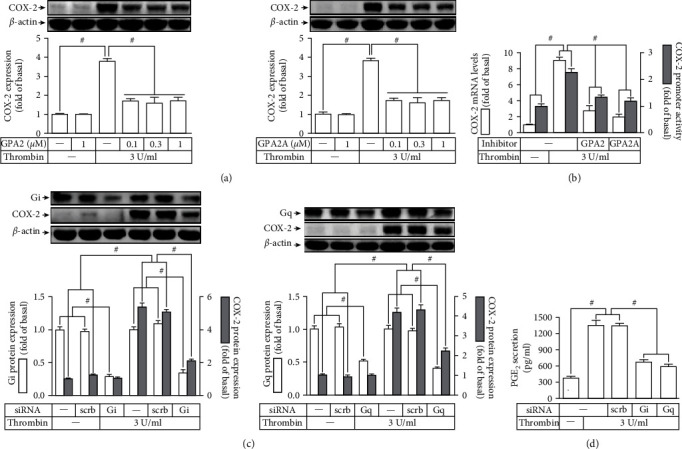
Thrombin enhances COX-2 expression via G-coupled receptor Gq and Gi/o subunit. (a) Cells were pretreated with GPA2 or GPA2A for 1 h and then incubated with thrombin for 6 h. The levels of COX-2 protein were determined. (b) Cells were pretreated with GPA2 (1 *μ*M) or GPA2A (1 *μ*M) for 1 h, and then incubated with thrombin for 4 h. The mRNA levels and promoter activity of COX-2 were determined. (c) Cells were transfected with scrambled, Gq*α*, or Gi*α* siRNA and then incubated with thrombin for 6 h. The levels of Gq*α*, or Gi*α*, and COX-2 protein were determined. (d) The media saved from (c) were used to determine the levels of PGE_2_ generation. Data are expressed as mean ± S.E.M. of three independent experiments. ^#^*P* < 0.05, as compared with the control or pretreatment with inhibitor indicated in the figure.

PARs are a subfamily of GPCRs. We more examined whether G_i/o_ or G_q_ protein-coupled receptor was engaged in the expression COX-2 induced by thrombin.  Figure 2(a) showed that pretreating HTSMCs with a G_i/o_ protein antagonist (GPA2) or a G_q_ protein antagonist (GPA2A) inhibited the expression of COX-2 generated by thrombin. In addition, GPA2 or GPA2A pretreatment also reduced the levels of mRNA and promoter activities of COX-2 stimulated by thrombin (Figure 2(b)). The effects of G_q_ and G_i/o_ in the thrombin-stimulated COX-2 upregulation were further ascertained by G_i_*α* or G_q_*α* siRNA. In this experiment, transfection with either G_i_*α* or G_q_*α* siRNA knocked down G_i_*α* or G_q_*α* protein expression and then markedly reduced the thrombin-enhanced COX-2 protein levels (Figure 2(c)). Moreover, transfection with these two siRNAs also reduced the release of PGE_2_ induced by thrombin (Figure 2(d)). Altogether, these results supported that G_q_ or G_i/o_ protein-coupled receptors mediate the thrombin-induced expression and secretion of COX-2/PGE_2_ in HTSMCs.

### 3.3. Thrombin Regulates Expression of COX-2 through PKC*α* Activation

**Figure 3:**
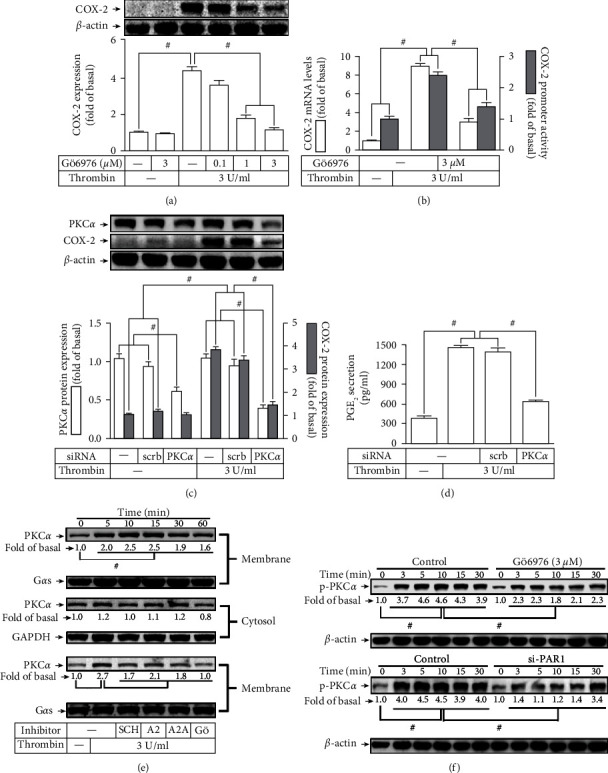
Thrombin induces COX-2 expression via PKC*α*. (a) Cells were pretreated with Gö6976 for 1 h and then incubated with thrombin for 6 h. The levels of COX-2 protein were determined. (b) Cells were pretreated with Gö6976 (3 *μ*M) for 1 h and then incubated with thrombin for 4 h. The mRNA levels and promoter activity of COX-2 were determined. (c) Cells were transfected with either scrambled or PKC*α* siRNA and then incubated with thrombin for 6 h. The levels of PKC*α* and COX-2 protein were determined. (d) The media saved from (c) were used to determine the levels of PGE_2_ generation. (e) Cells were treated with 3 U/ml thrombin for the indicated time intervals. The membrane and cytosolic fractions were prepared and subjected to Western blot using an anti-PKC*α* antibody. GAPDH and G*α*s were used as a marker protein for cytosolic and membrane fractions, respectively. (f) Cells were pretreated with SCH79797 (10 *μ*M), GPA2 (1 *μ*M), or GPA2A (1 *μ*M) for 1 h and then treated with thrombin for 10 min. The membrane fractions were prepared and subjected to Western blot using an anti-PKC*α* antibody. Data are expressed as mean ± S.E.M. of three independent experiments. ^#^*P* < 0.05, as compared with the control or pretreatment with inhibitor indicated in the figure.

Previous report indicated that in A549 cells, thrombin enhanced PKC*α* activity leading to protein expression [22]. In our recent report, PKC*α* has also been indicated to upregulate COX-2 induction [5]. Thus, we hypothesized that PKC*α* may engage in the COX-2 expression in HTSMCs triggered by thrombin.  Figure 3(a) showed that pretreatment of HTSMCs with PKC*α* inhibitor Gö6976 markedly diminished the COX-2 expression initiated by thrombin. In addition, as shown in Figure 3(b), Gö6976 also abrogated the mRNA levels and promoter activities of COX-2 induced by thrombin. To further verify that PKC*α* has an essential function in the thrombin-mediated COX-2 expression, the level of PKC*α* protein was knocked down by transfection with its siRNA which also significantly reduced the thrombin-enhanced protein levels of COX-2 (Figure 3(c)). Additionally, PKC*α* siRNA transfection also inhibited the thrombin-induced PGE_2_ secretion (Figure 3(d)). Besides, our discoveries proved that thrombin induced translocation of cytosolic PKC*α* into the membrane in a time-dependent manner (Figure 3(e)), which was blocked by SCH79797, GPA2, GPA2A, or Gö6976 (Figure 3(f)). Altogether, these discoveries indicated that PAR1/G_q_ or G_i/o_-dependent PKC*α* activation mediates the COX-2 upregulation leading to PGE_2_ synthesis induced by thrombin in HTSMCs.

### 3.4. Thrombin Regulates Expression of COX-2 through Activation of MAPKs

**Figure 4:**
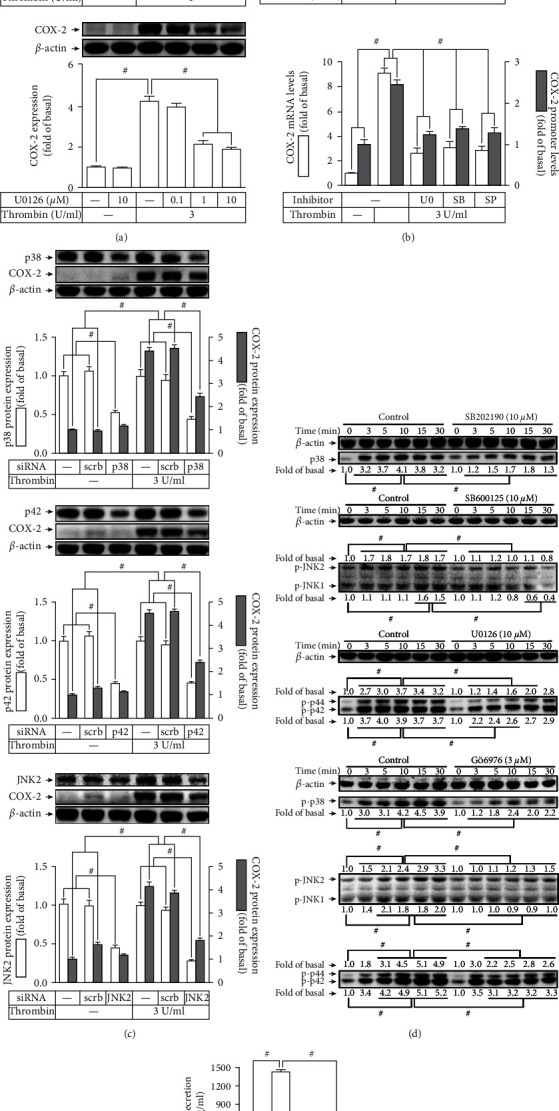
Thrombin induces COX-2 expression via MAPKs. (a) Cells were pretreated with SB202190, U0126, or SP600125 for 1 h and then incubated with thrombin for 6 h. The levels of COX-2 protein were determined by Western blot. (b) Cells were pretreated with U0126 (10 *μ*M), SB202190 (10 *μ*M), or SP600125 (10 *μ*M) for 1 h and then incubated with thrombin for 4 h. The mRNA levels and promoter activity of COX-2 were determined. (c) Cells were transfected with scrambled, p38, p42, or JNK2 siRNA and then incubated with thrombin for 6 h. The levels of p38, p42, JNK2, and COX-2 protein were determined. (d) Cells were pretreated without or with SB202190 (10 *μ*M), SP600125 (10 *μ*M), U0126 (10 *μ*M), or Gö6976 (3 *μ*M) for 1 h and then incubated with thrombin for the indicated time intervals. The levels of phospho-p38 MAPK, phospho-p42/p44 MAPK, and phospho-JNK1/2 were determined by Western blot. (e) The media saved from (a) were used to determine the levels of PGE2 generation. Data are expressed as mean ± S.E.M. of three independent experiments. ^#^*P* < 0.05, as compared with the control or pretreatment with inhibitor indicated in the figure.

Thrombin was proved to activate p38 MAPK [17], JNK1/2 [15], and p42/p44 MAPK [10, 16] in various types of cells. Moreover, MAPK activation-mediated COX-2 induction has been verified [5]. Thus, we hypothesized that MAPKs participated in the thrombin-induced upregulation of COX-2.  Figure 4(a) showed that pretreating HTSMCs with the inhibitor of p38 MAPK (SB202190), MEK1/2 (U0126), or JNK1/2 (SP600125) concentration-dependently inhibited the thrombin-induced COX-2 expression in HTSMCs. Additionally, as displayed in Figure 4(b), these inhibitors also reduced the levels of mRNA expression and promoter activities of COX-2 induced by thrombin. To further ensure that MAPKs have the critical roles in the thrombin-mediated responses, HTSMCs transfected with JNK2, p42, or p38 siRNA knocked down JNK2, p42, or p38 protein expression, individually, and then markedly inhibited the thrombin-enhanced the levels of COX-2 protein (Figure 4(c)). Besides, our results demonstrated that thrombin time-dependently enhanced phosphorylation of JNK1/2, p38 MAPK, and p42/p44 MAPK in HTSMCs, which were reduced by SP600125, SB202190, and U0126, individually (Figure 4(d)). It has been verified that PKC*α* can stimulate the activation of MAPKs in numerous types of cells [5, 22]. Thus, we investigated the relationship between MAPKs and PKC*α* activated by thrombin in HTSMCs. Here, we revealed that Gö6976 pretreatment significantly attenuated the thrombin-induced phosphorylation of p38 MAPK, JNK1/2, and p42/p44 MAPK in HTSMCs (Figure 4(d)). These findings implied that on the thrombin-stimulated responses, PKC*α* was the upstream signaling of MAPKs. Finally, pretreatment of HTSMCs with these three inhibitors of MAPKs also inhibited the thrombin-induced PGE_2_ release (Figure 4(e)). Altogether, these discoveries suggested that MAPK activation regulates the COX-2 upregulation associated with PGE_2_ release in HTSMCs exposed to thrombin.

### 3.5. Thrombin Induces Expression of COX-2 via NF-*κ*B

**Figure 5:**
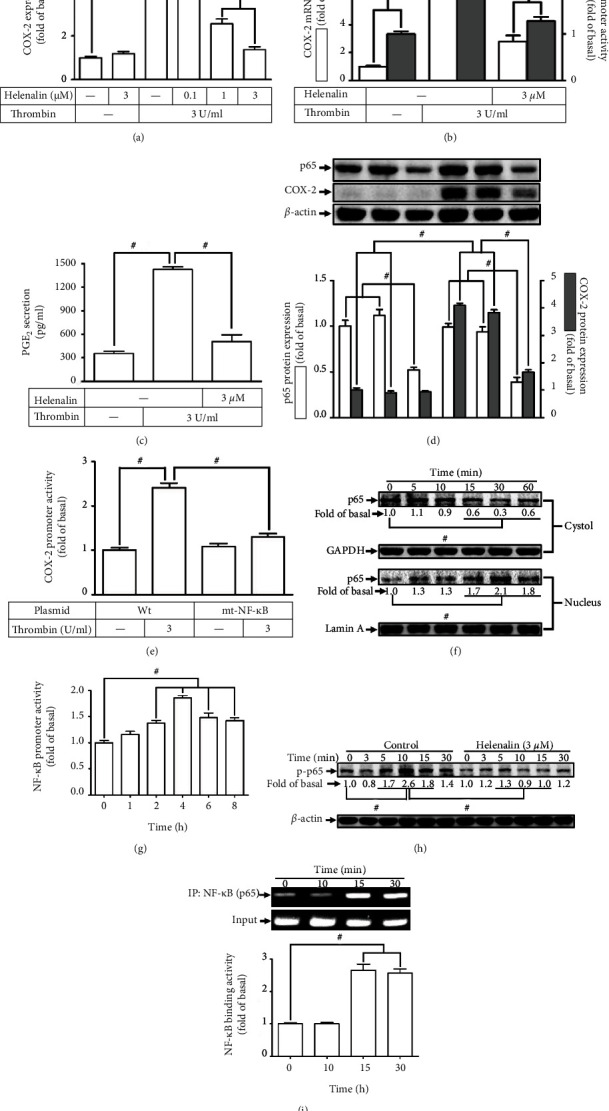
Thrombin induces COX-2 expression via NF-*κ*B. (a) Cells were pretreated with helenalin for 1 h and then incubated with thrombin for 6 h. The levels of COX-2 protein were determined by Western blot. (b) Cells were pretreated with helenalin (3 *μ*M) for 1 h and then incubated with thrombin for 4 h. The mRNA levels and promoter activity of COX-2 were determined. (c) The media saved from (a) were used to determine the levels of PGE2 generation. (d) Cells were transfected with either scrambled or p65 siRNA and then incubated with thrombin for 6 h. The levels of p65 and COX-2 protein were determined. (e) Cells were transfected with wild-type COX-2 promoter or mt-NF-*κ*B COX-2 promoter, and then incubated with thrombin for 4 h. The promoter activity of COX-2 was determined. (f) Cells were treated with thrombin for the indicated time intervals. The nuclear and cytosolic fractions were prepared and subjected to Western blot using an anti-p65 antibody. GAPDH and lamin A were used as a marker protein for cytosolic and nuclear fractions, respectively. (g) Cells were treated with thrombin for the indicated time intervals. The promoter activity of NF-*κ*B was determined. (h) Cells were pretreated without or with helenalin (3 *μ*M) for 1 h and then treated with thrombin for the indicated time intervals. The levels of phospho-p65 were determined by Western blot. (i) Cells were treated with thrombin for the indicated time intervals. The NF-*κ*B p65 binding activities were analyzed by a ChIP assay. Data are expressed as mean ± S.E.M. of three independent experiments. ^#^*P* < 0.05, as compared with the control or pretreatment with inhibitor indicated in the figure.

Khanal et al. have indicated that in RAW 264.7 cells, genipin-enhanced COX-2 protein expression is regulated by NF-*κ*B [4]. Thus, we hypothesized that NF-*κ*B participated in thrombin-induced COX-2 expression.  Figure 5(a) demonstrated that in HTSMCs, pretreating cells with helenalin (an inhibitor of NF-*κ*B) concentration dependently reduced thrombin-stimulated COX-2 protein expression. In addition, inhibition of NF-*κ*B by helenalin also repressed that the thrombin induced the levels of mRNA expression and promoter activities of COX-2 (Figure 5(b)) as well as secretion of PGE_2_ (Figure 5(c)). To ascertain the critical function of NF-*κ*B in the COX-2 expression induced by thrombin, the levels of p65 protein were knockdown in the cells transfected with p65 siRNA, which inhibited the protein level of COX-2 expression triggered by thrombin (Figure 5(d)). Besides, in HTSMCs, we showed that transfection with a point-mutated NF-*κ*B-COX-2 promoter prominently attenuated promoter activity of COX-2 stimulated by thrombin (Figure 5(e)). Further, we noticed that thrombin time-dependently promoted nuclear translocation of the cytosolic NF-*κ*B p65 and progressively increased promoter activity of NF-*κ*B, reaching a maximal reaction within 4 h (Figures 5(f) and 5(g)). We further investigated whether thrombin could induce phosphorylation of NF-*κ*B p65 in HTSMCs. Our results showed that thrombin time-dependently induced NF-*κ*B p65 activation with a maximal reaction within 10 min, which was abrogated by helenalin (Figure 5(h)). Moreover, our experiment adopted a ChIP assay to verify whether thrombin stimulates NF-*κ*B recruited into the promoter of COX-2 is an essential step in COX-2 upregulation.  Figure 5(i) showed that thrombin time-dependently recruited p65 to bind with the COX-2 promoter with a maximal binding reaction within 15-30 min. Altogether, these discoveries suggested that an NF-*κ*B-dependent pathway regulates the levels of COX-2 expression accompanied with PGE_2_ production in HTSMCs induced by thrombin.

### 3.6. Thrombin Induces Activation of NF-*κ*B via a PAR1/PKC*α*/MAPK Pathway

**Figure 6:**
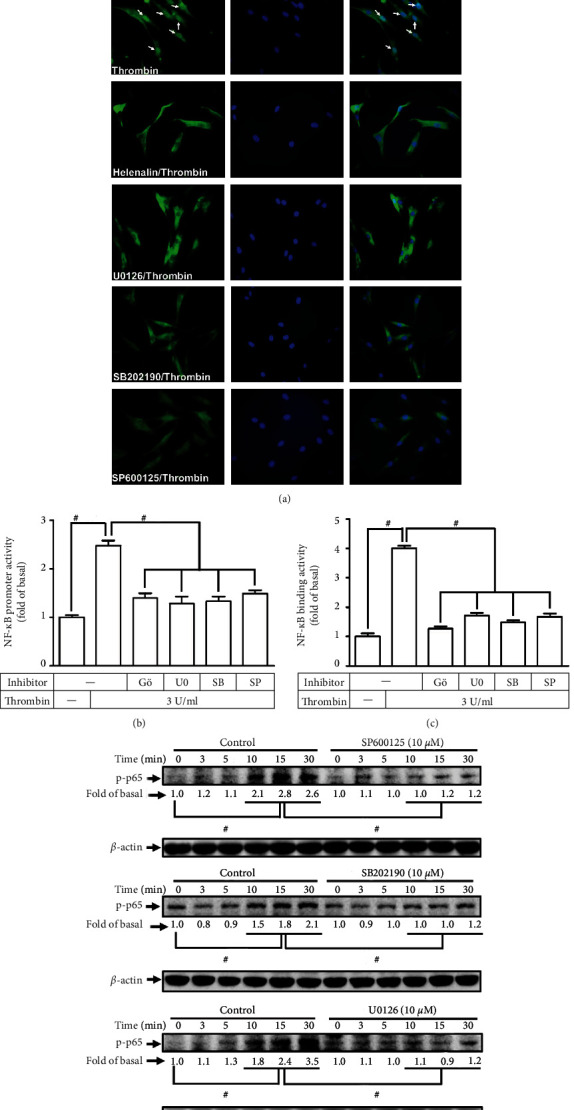
Thrombin induces NF-*κ*B activation via PKC*α*/MAPKs. (a) Cells were pretreated with helenalin (3 *μ*M), U0126 (10 *μ*M), SB202190 (10 *μ*M), or SP600125 (10 *μ*M) for 1 h and then incubated with thrombin for 30 min. Cells were fixed and then labeled with an anti-phospho-p65 antibody and then FITC-conjugated secondary antibody. Individual cells were imaged. Scale bar: 50 *μ*m. (b) Cells were pretreated with Gö6976 (3 *μ*M), U0126 (10 *μ*M), SB202190 (10 *μ*M), or SP600125 (10 *μ*M) for 1 h and then incubated with thrombin for 4 h. The promoter activity of NF-*κ*B was determined. (c) Cells were pretreated with Gö6976 (3 *μ*M), U0126 (10 *μ*M), SB202190 (10 *μ*M), or SP600125 (10 *μ*M) for 1 h and then incubated with thrombin for 30 min. The NF-*κ*B p65 binding activities were analyzed by a ChIP assay. (d) Cells were pretreated without or with SP600125 (10 *μ*M), SB202190 (10 *μ*M), or U0126 (10 *μ*M) for 1 h, and then incubated with thrombin for the indicated time intervals. The levels of phospho-p65 were determined by Western blot. Data are expressed as mean ± S.E.M. of three independent experiments. ^#^*P* < 0.05, as compared with the control or pretreatment with inhibitor indicated in the figure.

A variety of signaling components, PI3K/Akt [23], PKC*α* [23, 24], and MAPKs [5, 20], have been proved to stimulate NF-*κ*B activity. Here, in HTSMCs, we investigated the relationship of NF-*κ*B, PKC*α*, and MAPKs in response to thrombin. First, we revealed that pretreating HTSMCs with helenalin, U0126, SB202190, or SP600125 significantly blocked the thrombin-stimulated cytosolic NF-*κ*B p65 translocation into the nucleus, examined by immunofluorescent staining (Figure 6(a)). Additionally, the thrombin-stimulated promoter activity of NF-*κ*B and the p65 binding ability with the promoter of COX-2 were impeded by Gö6976, SB202190, SP600125, or U0126 (Figures 6(b) and 6(c)). Finally, our data demonstrated that thrombin-induced phosphorylation of NF-*κ*B p65 was impeded by SB202190, SP600125, or U0126 (Figure 6(d)). Therefore, we suggested that thrombin-stimulated NF-*κ*B activation is regulated by a PAR1/G_q_ or G_i/o_/PKC*α*/MAPKs pathway in HTSMCs.

## 4. Discussion

**Figure 7:**
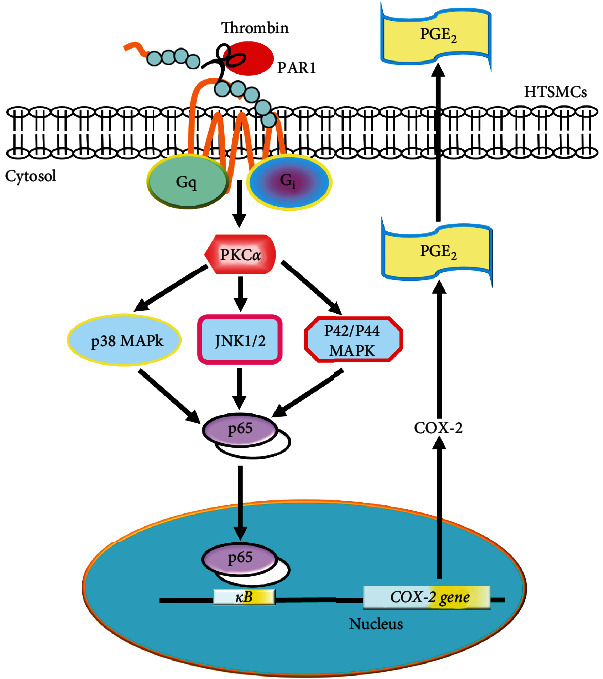
Schematic diagram illustrating the proposed signaling pathway involved in thrombin-induced COX-2 expression and PGE_2_ secretion in HTSMCs. Thrombin-induced COX-2 expression associated with PGE_2_ generation was mediated through PAR1/G_q_ or G_i/o_/PKC*α*/MAPK-dependent NF-*κ*B activation in HTSMCs.

Thrombin-triggered responses in cells are predominantly mediated by the family of receptors PARs, although a few exceptions have been observed [21]. A previous study indicated that thrombin can promote airway remodeling via PAR1 [25]. Immunoreactive PARs have been demonstrated in various types of cells in the respiratory airways. PAR activation has been shown to stimulate cell mitogenesis and induce tissue inflammation [26]. Endogenous PGE_2_ has been uncovered to be able to regulate and maintain the spontaneous trachea tone of guinea pigs through activation of contractile receptors of EP1 and relaxant receptors of EP2 [27]. Therefore, PGE_2_ can mediate bronchodilation and anti-inflammatory effects via the EP2 and/or EP4 receptors [28]. In contrast, PGE_2_ has been proved to be higher levels in lung fibroblasts of COPD compared with control subjects. PGE_2_ can induce fibroblast senescence and related inflammation accompanied with COX-2-dependent production of reactive oxygen species and activation of p53 *in vitro and in vivo* [29]. Moreover, PGE_2_ also contributes as a mediator of proinflammation and angiogenesis within the airways of COPD subjects via the increased expression of interleukin-8 and vascular endothelial growth factor [30]. Previous study also supports that high levels of COX-2-synthesized PGE_2_ participate in airway inflammation [31]. Chronic inflammatory states related to COX-2 upregulation may be an important step towards lung cancer [32]. Thus, COX-2-derived PGE_2_ could be a crucial factor in respiratory inflammatory diseases. However, in HTSMCs, the molecular mechanisms underlying thrombin-induced COX-2 expression are not completely characterized. In this research, we verified that transfection with siRNA of PAR1, G_q_*α*, G_i_*α*, PKC*α*, p38, JNK2, p42, or p65 for specific gene silencing and the inhibitors of PAR1, G_i/o_, G_q_, PKC*α*, MEK1/2, p38 MAPK, JNK1/2, or NF-*κ*B pretreatment reduced the levels of COX-2 upregulation associated with PGE_2_ synthesis stimulated by thrombin (Figure 7). This study established that the COX-2 expression was mediated through PAR1/G_i/o_ or G_q_/PKC*α*/MAPK-dependent NF-*κ*B activation in HTSMCs challenged with thrombin.

Thrombin is well recognized for its pivotal effects in the maintenance of homeostasis, which is a procoagulant serine protease, released during the initial stage of intravascular coagulation related to injury of tissue. On the other hand, thrombin also stimulates a broad range of cellular responses including many reactions of immune and inflammation [6]. Asero et al. revealed higher levels of plasma prothrombin fragments 1 + 2 in asthma subjects (267 ± 243 pM) than normal volunteers, suggesting that hyperactivation of the coagulation reaction can induce the production of the proinflammatory cytokine involved in the pathologic process of asthma [33]. Another report also unveiled that asthma patients had a higher maximal prothrombin conversion rate and higher thrombin concentration compared with controls [8]. In COPD patients, maximum thrombin levels were higher than in controls, as were factor II levels and rates of thrombin generation [7]. GPCRs constitute a large family of receptors responsible for activating intracellular signal transduction pathways while it senses molecules outside the cells, ultimately, leading to cellular responses. Thrombin has been disclosed to play direct effects on the cells through activation of PARs, a family of GPCRs [6, 21]. Up to now, four isoforms known as PAR1-4 have been identified. Previous studies suggested that thrombin possesses many important effects in the inflammatory reactions in the respiratory system, causing the accumulation of neutrophils in the airway and increasing the levels of tumor necrosis factor- (TNF-) *α* in BAL fluid in mice challenged with thrombin [34]. Moreover, thrombin stimulating lung epithelial cells acting through PAR1 and PAR4 but not PAR-3 can induce the upregulation and secretion of IL-8, PGE_2_, and IL-6 [35]. Therefore, thrombin appears to be a conventional pathogen in various inflammatory diseases of the airway and lung. Indeed, we found that PAR1-3 exist on the cell membrane of HTSMCs. In this research, we verified that thrombin potentially induced COX-2 upregulation associated with PGE_2_ secretion via PAR1/G_i/o_ or G_q_ which was validated by transfecting or pretreating HTSMCs with their respective siRNAs or pharmacological inhibitors. Thus, we suggested that these reactions caused by thrombin/PAR1/G_i/o_ or G_q_ might promote airway inflammation. In the future, we will investigate whether PAR3 also participates in thrombin-induced airway inflammation.

PKCs regulate a variety of pathways involved in cell stress responsiveness, growth, and death. The isoforms of PKCs are divided into three categories [36]. Moreover, Chen et al. previously indicated that in human airway epithelial (NCI-H292) cells, TNF-*α* upregulates COX-2 expression accompanied with PGE_2_ release via activation of PKC*α* [37]. Additionally, the previous study also disclosed that thrombin could induce PKC*α* activation, leading to epithelial-mesenchymal transition and collagen I secretion in A549 human alveolar epithelial cells [22]. PKC*α* involved is revealed by our findings indicating that genetic silencing or pharmacological inhibitor of PKC*α* significantly inhibited the levels of COX-2 upregulation associated with PGE_2_ secretion in HTSMCs exposed to thrombin. Thus, our results in HTSMCs suggested that upregulation of COX-2 associated with PGE_2_ secretion by thrombin are mediated via pathways dependent on PKC*α* activation.

MAPKs are activated by various stimuli and crucial signaling modules. MAPKs consist of three groups which have been revealed as p42/p44 MAPK, p38 MAPK, and JNK1/2. Thrombin has also been disclosed to induce activation of JNK1/2 [15], p42/p44 MAPK [10, 16], and p38 MAPK [17] in various types of cells. MAPKs have also been revealed to promote COX-2 expression [5], consistent with our findings showing that, in HTSMCs, genetic silencing and pretreating with the inhibitors of p38 MAPK, JNK1/2, and p42/p44 MAPK reduced the levels of COX-2 expression and PGE_2_ synthesis induced by thrombin. Therefore, we suggested that MAPKs exert vital effects in mediating the induction of COX-2 expression and PGE_2_ synthesis by thrombin in HTSMCs. MAPK activation is regulated by PKC*α* in a variety of types of cells [22, 38]. Moreover, in HTSMCs, PKC*α* inhibition could reduce the thrombin-induced MAPK phosphorylation. Therefore, we established that thrombin enhanced the levels of COX-2 expression and PGE_2_ synthesis via PAR1/PKC*α*-dependent MAPK activation in HTSMCs.

Previous studies discovered that the COX-2 promoter contains numerous binding sites of functional activator elements, including AP-1 and NF-*κ*B [5]. NF-*κ*B possesses a conventional role in the evolution phase of inflammation and thus is regarded as the main regulator of inflammatory reactions. A variety of factors, including cytokines, thrombin, chemical, and physical stresses, and viral and bacterial infections could enhance NF-*κ*B activation [18–20, 39]. However, in HTSMCs, NF-*κ*B participated in the thrombin-induced COX-2 expression remains unclear. Our findings displayed that the levels of COX-2 expression triggered by thrombin were significantly inhibited by pretreatment with an NF-*κ*B inhibitor helenalin or p65 siRNA transfection. Additionally, transfection with the COX-2 promoter contained a point-mutated binding site of NF-*κ*B and inhibited the COX-2 promoter activity stimulated by thrombin, indicating that NF-*κ*B activity was enhanced by thrombin and involved in the upregulation of COX-2 levels in HTSMCs. Moreover, we demonstrated that thrombin enhanced phosphorylation and translocation of NF-*κ*B p65, the binding of p65 to the COX-2 promoter, and NF-*κ*B promoter activity as well, which was mitigated by Gö6976, SP600125, SB202190, or U0126. Consistently, our results revealed that thrombin enhanced the levels of COX-2 expression associated with PGE_2_ synthesis via a PAR1/PKC*α*/MAPKs/NF-*κ*B pathway in HTSMCs.

## 5. Conclusions

In this report in HTSMCs, we constructed that thrombin-enhanced levels of COX-2 expression associated with PGE_2_ synthesis were mediated via PAR1/G_q_ or G_i/o_/PKC*α*/MAPK-dependent NF-*κ*B activation (Figure 7). This report provides new visions into the mechanisms of thrombin-stimulated upregulation of COX-2 and PGE_2_ release, leading to an exaggeration of the inflammatory reaction. A better understanding of mechanisms underlying the regulation of the gene of *COX-2* will establish more opportunities to develop anti-inflammatory therapeutic strategies for treating lung inflammation.

## Data Availability

The data used to support the findings of this study are available from the corresponding author upon request.

## Abbreviations

AA: Arachidonic acid
BSA: Bovine serum albumin
ChIP: Chromatin immuno-precipitation
COX-2: Cyclooxygenase-2
DMEM: Dulbecco's modified Eagle's medium
ECL: Enhanced chemiluminescence
ELISA: Enzyme-linked immunosorbent assay
GAPDH: Glyceraldehyde-3-phosphate dehydrogenase
GPA2: GP antagonist-2
GPA2A: GP antagonist-2A
GPCRs: G-protein-coupled receptors
HTSMCs: Human tracheal smooth muscle cells
IgG: Immunoglobulin G
JNK: c-Jun N-terminal kinase
MAPK: Mitogen-activated protein kinase
PAR: Protease-activated receptor
PBS: Phosphate-buffered saline
PGE_2_: Prostaglandin E_2_
PKC: Protein kinase C
SDS-PAGE: Sodium dodecyl sulfate polyacrylamide gel electrophoresis
siRNA: Small interfering RNA
Tris-HCl: Tris [hydroxymethyl]aminomethane
TTBS: Tween-Tris-buffered saline.

## References

[1] Lam M., Lamanna E., Bourke J. E. (2019). Regulation of airway smooth muscle contraction in health and disease. *Advances in Experimental Medicine and Biology*.

[2] Mastalerz L., Celejewska-Wójcik N., Wójcik K. (2015). Induced sputum supernatant bioactive lipid mediators can identify subtypes of asthma. *Clinical and Experimental Allergy*.

[3] Lee I. T., Lee C. W., Tung W. H. (2010). Cooperation of TLR2 with MyD88, PI3K, and Rac1 in lipoteichoic acid-induced cPLA_2_/COX-2-dependent airway inflammatory responses. *The American Journal of Pathology*.

[4] Khanal T., Kim H. G., Do M. T. (2014). Genipin induces cyclooxygenase-2 expression via NADPH oxidase, MAPKs, AP-1, and NF-*κ*B in RAW 264.7 cells. *Food and Chemical Toxicology*.

[5] Yang C. C., Hsiao L. D., Su M. H., Yang C. M. (2020). Sphingosine 1-phosphate induces cyclooxygenase-2/prostaglandin E_2_ expression via PKC*α*-dependent mitogen-activated protein kinases and NF-*κ*B cascade in human cardiac fibroblasts. *Frontiers in Pharmacology*.

[6] Siller-Matula J. M., Schwameis M., Blann A., Mannhalter C., Jilma B. (2011). Thrombin as a multi-functional enzyme. Focus on in vitro and in vivo effects. *Thrombosis and Haemostasis*.

[7] Undas A., Jankowski M., Kaczmarek P., Sladek K., Brummel-Ziedins K. (2011). Thrombin generation in chronic obstructive pulmonary disease: dependence on plasma factor composition. *Thrombosis Research*.

[8] Bazan-Socha S., Mastalerz L., Cybulska A. (2016). Asthma is associated with enhanced thrombin formation and impaired fibrinolysis. *Clinical and Experimental Allergy*.

[9] Wygrecka M., Didiasova M., Berscheid S. (2013). Protease-activated receptors (PAR)-1 and -3 drive epithelial-mesenchymal transition of alveolar epithelial cells - potential role in lung fibrosis. *Thrombosis and Haemostasis*.

[10] Shih C. H., Bien M. Y., Chiang L. L., Su C. L., Lin C. H., Chen B. C. (2009). Thrombin induces cyclooxygenase-2 expression via the ERK and NF-*κ*B pathways in human lung fibroblasts. *European Journal of Pharmacology*.

[11] Altieri P., Bertolotto M., Fabbi P. (2018). Thrombin induces protease-activated receptor 1 signaling and activation of human atrial fibroblasts and dabigatran prevents these effects. *International Journal of Cardiology*.

[12] Sakai H., Yamamoto M., Chiba Y., Misawa M. (2009). Some different effect of PKC inhibitors on the acetylcholine, and endothelin-1-induced contractions of rat bronchial smooth muscle. *European Journal of Pharmacology*.

[13] Widmann C., Gibson S., Jarpe M. B., Johnson G. L. (1999). Mitogen-activated protein kinase: conservation of a three-kinase module from yeast to human. *Physiological Reviews*.

[14] Athari S. S. (2019). Targeting cell signaling in allergic asthma. *Signal Transduction and Targeted Therapy*.

[15] Miho N., Ishida T., Kuwaba N. (2005). Role of the JNK pathway in thrombin-induced ICAM-1 expression in endothelial cells. *Cardiovascular Research*.

[16] Lin C. H., Nai P. L., Bien M. Y., Yu C. C., Chen B. C. (2014). Thrombin-induced CCAAT/enhancer-binding protein *β* activation and IL-8/CXCL8 expression via MEKK1, ERK, and p90 ribosomal S6 kinase 1 in lung epithelial cells. *Journal of Immunology*.

[17] Kanda Y., Watanabe Y. (2005). Thrombin-induced glucose transport via Src-p38 MAPK pathway in vascular smooth muscle cells. *British Journal of Pharmacology*.

[18] Mitchell J. P., Carmody R. J. (2018). NF-*κ*B and the transcriptional control of inflammation. *International Review of Cell and Molecular Biology*.

[19] Lawrence T. (2009). The nuclear factor NF-kappaB pathway in inflammation. *Cold Spring Harbor Perspectives in Biology*.

[20] Fang X., Liao R., Yu Y., Li J., Guo Z., Zhu T. (2019). Thrombin induces secretion of multiple cytokines and expression of protease-activated receptors in mouse mast cell line. *Mediators of Inflammation*.

[21] Coughlin S. R. (1999). How the protease thrombin talks to cells. *Proceedings of the National Academy of Sciences of the United States of America*.

[22] Song J. S., Kang C. M., Park C. K., Yoon H. K. (2013). Thrombin induces epithelial-mesenchymal transition via PAR-1, PKC, and ERK1/2 pathways in A549 cells. *Experimental Lung Research*.

[23] Zhang Y., Cardell L. O., Edvinsson L., Xu C. B. (2013). MAPK/NF-*κ*B-dependent upregulation of kinin receptors mediates airway hyperreactivity: a new perspective for the treatment. *Pharmacological Research*.

[24] Zheng J., Kong C., Yang X., Cui X., Lin X., Zhang Z. (2017). Protein kinase C-*α* (PKC*α*) modulates cell apoptosis by stimulating nuclear translocation of NF-kappa-B p65 in urothelial cell carcinoma of the bladder. *BMC Cancer*.

[25] Zhu W., Bi M., Liu Y. (2013). Thrombin promotes airway remodeling via protease-activated receptor-1 and transforming growth factor-*β*1 in ovalbumin-allergic rats. *Inhalation Toxicology*.

[26] Lan R. S., Stewart G. A., Henry P. J. (2002). Role of protease-activated receptors in airway function: a target for therapeutic intervention?. *Pharmacology & Therapeutics*.

[27] Säfholm J., Dahlén S. E., Delin I. (2013). PGE_2_ maintains the tone of the guinea pig trachea through a balance between activation of contractile EP_1_ receptors and relaxant EP_2_ receptors. *British Journal of Pharmacology*.

[28] Sastre B., del Pozo V. (2012). Role of P G E 2 in asthma and nonasthmatic eosinophilic bronchitis. *Mediators of Inflammation*.

[29] Dagouassat M., Gagliolo J. M., Chrusciel S. (2013). The cyclooxygenase-2-prostaglandin E_2_ pathway maintains senescence of chronic obstructive pulmonary disease fibroblasts. *American Journal of Respiratory and Critical Care Medicine*.

[30] Bonanno A., Albano G. D., Siena L. (2016). Prostaglandin E_2_ possesses different potencies in inducing vascular endothelial growth factor and interleukin-8 production in COPD human lung fibroblasts. *Prostaglandins, Leukotrienes, and Essential Fatty Acids*.

[31] Park G. Y., Christman J. W. (2006). Involvement of cyclooxygenase-2 and prostaglandins in the molecular pathogenesis of inflammatory lung diseases. *American Journal of Physiology. Lung Cellular and Molecular Physiology*.

[32] Martey C. A., Pollock S. J., Turner C. K. (2004). Cigarette smoke induces cyclooxygenase-2 and microsomal prostaglandin E_2_ synthase in human lung fibroblasts: implications for lung inflammation and cancer. *American Journal of Physiology. Lung Cellular and Molecular Physiology*.

[33] Asero R., Tedeschi A., Cugno M. (2011). Markers of autoreactivity, coagulation and angiogenesis in patients with nonallergic asthma. *Allergy*.

[34] Moffatt J. D., Lever R., Page C. P. (2004). Effects of inhaled thrombin receptor agonists in mice. *British Journal of Pharmacology*.

[35] Asokananthan N., Graham P. T., Fink J. (2002). Activation of protease-activated receptor (PAR)-1, PAR-2, and PAR-4 stimulates IL-6, IL-8, and prostaglandin E_2_ release from human respiratory epithelial cells. *Journal of Immunology*.

[36] Wu-Zhang A. X., Newton A. C. (2013). Protein kinase C pharmacology: refining the toolbox. *The Biochemical Journal*.

[37] Chen C. C., Sun Y. T., Chen J. J., Chiu K. T. (2000). TNF-*α*-induced cyclooxygenase-2 expression in human lung epithelial cells: involvement of the phospholipase C-*γ*2, protein kinase C-*α*, tyrosine kinase, NF-*κ*B-inducing kinase, and I-*κ*B kinase 1/2 pathway. *Journal of Immunology*.

[38] Hill K. S., Erdogan E., Khoor A. (2014). Protein kinase C*α* suppresses *Kras* -mediated lung tumor formation through activation of a p38 MAPK-TGF*β* signaling axis. *Oncogene*.

[39] Karin M., Greten F. R. (2005). NF-*κ*B: linking inflammation and immunity to cancer development and progression. *Nature Reviews Immunology*.

